# Relation is an option for processing context information

**DOI:** 10.3389/frai.2022.924688

**Published:** 2022-10-11

**Authors:** Kazunori D Yamada, M. Samy Baladram, Fangzhou Lin

**Affiliations:** ^1^Unprecedented-scale Data Analytics Center, Tohoku University, Sendai, Japan; ^2^Graduate School of Information Sciences, Tohoku University, Sendai, Japan; ^3^Artificial Intelligence Research Center, National Institute of Advanced Industrial Science and Technology, Tokyo, Japan

**Keywords:** Attention, artificial intelligence, neural networks, multilayer perceptron, Transformer, time complexity, Relation

## Abstract

Attention mechanisms are one of the most frequently used architectures in the development of artificial intelligence because they can process contextual information efficiently. Various artificial intelligence architectures, such as Transformer for processing natural language, image data, etc., include the Attention. Various improvements have been made to enhance its performance since Attention is a powerful component to realize artificial intelligence. The time complexity of Attention depends on the square of the input sequence length. Developing methods to improve the time complexity of Attention is one of the most popular research topics. Attention is a mechanism that conveys contextual information of input sequences to downstream networks. Thus, if one wants to improve the performance of processing contextual information, the focus should not be confined only on improving Attention but also on devising other similar mechanisms as possible alternatives. In this study, we devised an alternative mechanism called “Relation” that can understand the context information of sequential data. Relation is easy to implement, and its time complexity depends only on the length of the sequences; a comparison of the performance of Relation and Attention on several benchmark datasets showed that the context processing capability of Relation is comparable to that of Attention but with less computation time. Processing contextual information at high speeds would be useful because natural language processing and biological sequence processing sometimes deal with very long sequences. Hence, Relation is an ideal option for processing context information.

## 1. Introduction

Attention is a mechanism developed in 2017 to reveal the relationship between different positions in sequential data (Vaswani et al., 2017). The basic principles of Attention have been implemented in numerous research fields, and it has exhibited outstanding performance to date. Its performance has been particularly successful in the field of natural language processing, where many pretrained models such as bidirectional encoder representations from transformers (BERT), are built based on Attention.

Recurrent neural networks (RNNs), such as Long Short-Term Memory and Gated Recurrent Unit, were mainly used to process sequence data (Hochreiter and Schmidhuber, 1997; Cho et al., 2014) before the advent of Attention. One of the advantages of Attention over RNNs is its computational speed. RNNs, due to their structure, process the tokens of a sequence in a sequential manner, which cannot fully utilize parallel computing. Therefore, abundant computing resources cannot be utilized efficiently. However, sequence processing using Attention can be parallelized, thus allowing complete utilization of computing resources. Since the size of data handled by deep learning methods is very large, the efficiency of the computation is an important indicator for architecture selection.

This study focused on self-Attention, which is used to calculate the relationship between tokens in a single sequence (Shim et al., 2022). The time complexity of Attention is *O*(*N*^2^) when the length of the input sequence is *N*. The computation time required to calculate the square of *N* can be troublesome since real-world data are sometimes lengthy. Therefore, many attempts have been made from various perspectives to reduce the amount of time complexity (Tay et al., 2020). Among the methods developed to date, Linear Attention provides the best time complexity in which the value is reduced to *O*(*N*) (Katharopoulos et al., 2020).

Attention, including Linear Attention, is a very powerful method for processing contextual information in sequential data. However, according to the universal approximation theorem, any multilayer perceptron (MLP) with sufficient expressive power can approximate any nonlinear function in the real world even without complex architectures, such as RNNs, convolutional neural networks (CNNs), and attention neural network (Cybenko, 1989). In other words, the attention mechanism is not necessarily an essential structure for artificial intelligence to understand contextual information. This is indicated in a previous study as well (Tolstikhin et al., 2021). The MLP is calculated for every token when employing Attention for contextual processing; this structure is called point-wise MLP. This point-wise computation is done for each token; however, it does not convey the relationship between tokens in sequences to the downstream network. Hence, Attention is used for adding information between tokens in input sequences to the point-wise MLP. The context needs to efficiently convey the contextual information of the entire input sequence to the downstream network for a neural network to efficiently process and understand it. Conversely, it is possible to employ alternative methods without using Attention if each point-wise MLP can appropriately add contextual information derived from the entire input sequence.

In this study, we have devised an alternative method called Relation for conveying contextual information to each point-wise MLP. It is a simple structure that conveys contextual information from an input sequence to each point-wise MLP. The motivation behind developing the proposed method was to improve the computation time for Attention. Relation improves the time complexity by avoiding the matrix product calculation that generates a matrix of size *N*×*N* while calculating Attention. The time complexity of Relation is *O*(*N*), which is the same as that of Linear Attention. Additionally, we analyzed if Relation can replace Attention and Linear Attention using several well-known natural language processing benchmark datasets. When comparing computation speeds on these benchmark datasets, we observed that the computation speed of Relation was significantly faster than that of Attention and Linear Attention while maintaining a comparable degree of prediction performance.

## 2. Related work

The field of computer vision has employed attention mechanism for a long time. The first study to use it for context processing was conducted in 2017 (Vaswani et al., 2017). The attention mechanism was used to implement the encoder-decoder model, a model used earlier for machine translation and building dialogue agents. The authors named this attention-based encoder-decoder model Transformer. Transformer has been used in various fields to solve a variety of problems. While the aforementioned BERT is a research achievement within the domain of natural language processing, for example, Vision Transformer (Dosovitskiy et al., 2021) and Image Transformer (Parmar et al., 2018) are applications of Transformer in the field of computer vision. Vision Transformer is almost the same as the original Transformer, and Image Transformer is a Transformer that incorporates techniques used in CNNs, a network architecture commonly used in computer vision. The development of these Transformers is an example of applied research on the attention mechanism. However, as was previously indicated, research has also been conducted from another perspective to reduce the computational complexity of Attention (Tay et al., 2020). Sparse Transformer (Child et al., 2019) improves the computational complexity to $O\left(N\sqrt{N}\right)$. Furthermore, Reformer (Kitaev et al., 2020) and Cluster-Former (Wang et al., 2021) have improved the computational complexity to *O*(*N*log*N*). Recently, methods have been developed that achieve linear computational complexity with respect to the sequence length. Linear Attention was one of the pioneer methods to achieve linear computational complexity by a simple modification of the computation of attention mechanism, where the order of the matrix multiplications required in the process of computing Attention was modified, as described in the following sections. Since then, research on reducing the computational complexity of attention mechanism has continued, and methods such as Performer (Choromanski et al., 2021), Linformer (Wang et al., 2020), Random Feature Attention (Peng et al., 2021), and other methods have achieved linear computational complexity of attention mechanism with similar prediction performance to Linear Attention.

## 3. Method

### 3.1. Attention and Relation

#### 3.1.1. Attention and Linear Attention

Here, we have demonstrated the computation of Attention *A* and Linear Attention *L* for the input sequence *x*∈ℝ^*N*×*m*^. The length of the input sequence is *N*, and let *m* be the size of the feature vector of each token in the input sequence. Initially, the following equations are used to compute the query, key, and value matrices:


(1) $$\begin{array}{lll} Q & = & xW_{Q}, \end{array}$$

(2) $$\begin{array}{lll} K & = & xW_{K}, \end{array}$$

(3) $$\begin{array}{lll} V & = & xW_{V}, \end{array}$$

where $W_{Q}\in {\mathbb{R}}^{m\times d}$, $W_{K}\in {\mathbb{R}}^{m\times d}$, and $W_{V}\in {\mathbb{R}}^{m\times d}$ are the *m*×*d* of the trainable parameter matrix that is responsible for projecting each token of the input sequence into a vector of *d* elements, where *d* is the size of Attention and is called depth.

Attention *A* is calculated using the following equation:


(4) $$\begin{array}{l} A\left(x\right)=\sigma \left(\frac{QK^{T}}{\sqrt{d}}\right)V, \end{array}$$

where σ is the softmax function, applied row-wise to *QK*^T^. The time complexity of Attention is *O*(*N*^2^) because it includes multiplication between the *N*×*d* matrix and *d*×*N*, generating the *N*×*N* matrix.

Next, Linear Attention *L* is computed by the following equation:


(5) $$\begin{array}{l} L\left(x\right)=\tau \left(Q\right)\left(\tau {\left(K\right)}^{T}V\right), \end{array}$$

where τ is defined by the following equation:


(6) $$\tau (x)=\{\begin{array}{ll} x+1 & \left(x>0\right)\\ e^{x} & \left(x\leq 0\right) \end{array}$$

In the computation of Linear Attention, the *d*×*N* matrix and the *N*×*d* matrix are first computed, resulting in the *d*×*d* matrix, and then the *N*×*d* matrix and the *d*×*d* matrix are multiplied. Therefore, the time complexity of Linear Attention is *O*(*N*) (Katharopoulos et al., 2020).

#### 3.1.2. Relation

In this study, we have devised a method called Relation, which conveys the entire contextual information of the input sequence to the downstream point-wise MLP without the use of Attention. First, *G* and *H* are generated using the following equations:


(7) $$\begin{array}{lll} G & = & xW_{G}, \end{array}$$

(8) $$\begin{array}{lll} H & = & xW_{H}, \end{array}$$

where $W_{G}\in {\mathbb{R}}^{m\times d}$ and $W_{H}\in {\mathbb{R}}^{m\times d}$ are trainable parameter matrices in *m*×*d* where each token in the input sequence is projected to a vector of *d* elements. Each row of *H* is considered a vector as follows:


(9) $$\begin{array}{l} H={\left[\begin{array}{cccc} h_{1} & h_{2} & \cdots & h_{N} \end{array}\right]}^{T}. \end{array}$$

Next, *h*′ is computed from the element-wise mean of *h*_*i*_ using the following formula:


(10) $$\begin{array}{l} h^{\prime }=\frac{1}{N}\overset{N}{\underset{i=1}{\sum }}h_{i}. \end{array}$$

Let *H*′ be the matrix of *N* rows, each row of which is the vector *h*′:


(11) $$\begin{array}{l} H^{\prime }={\left[\begin{array}{cccc} h^{\prime } & h^{\prime } & \cdots & h^{\prime } \end{array}\right]}^{T}\in {\mathbb{R}}^{N\times d} \end{array}$$

Finally, the Relation *R* is calculated using the trainable parameter matrix *W*∈ℝ^*d*×*d*^ as follows:


(12) $$\begin{array}{l} R\left(x\right)=\phi \left(\left(G⊙H^{\prime }\right)W\right) \end{array}$$

where ⊙ is the operator for computing the Hadamard product, and ϕ is a nonlinear activation function such as the rectified linear unit.

**Figure 1:**
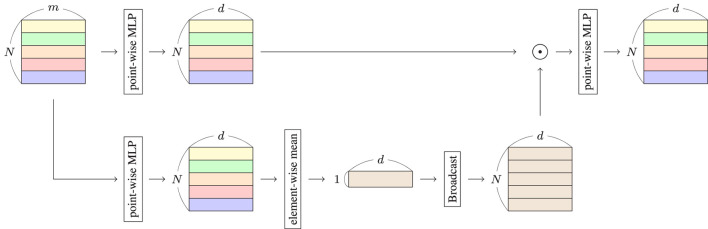
Structure of relation system. Input data are in the form of a matrix ∈ℝ^*N*×*m*^, where the length and feature vector size of input data are *N* and *m*, respectively. Output data are in the form of a matrix ∈ℝ^*N*×*d*^, where *d* is the depth of the Relation system.

In this Relation system, *h*′ is the data containing the features of the entire input sequence. We have interpreted each row of *G* as a weight that allows each token in the input sequence to extract information from *h*′. The overall view of the Relation system is shown in Figure 1. As shown in the figure, the input data ∈ℝ^*N*×*m*^ are projected to *G* by a point-wise MLP, which is used to compute the Hadamard product with *H*′. In the bottom panel, *H* is generated from the input data. The mean of elements in each row of *H* is calculated to generate *h*′. Subsequently, *h*′ is broadcasted to *H*′. Finally, Relation *R* is computed from the Hadamard product using point-wise MLP. The maximum size of the generated matrices in the computation is *N*×*d*, implying the time complexity of Relation is *O*(*N*).

### 3.2. Benchmark

#### 3.2.1. Network architecture

We used a simple structure without piling up the layers for all the benchmarks conducted in the study so that we could compare the performance of Attention and Relation without the influence of other complex factors. In all network structures, either Attention or Relation was computed from the input sequence followed by a single layer of point-wise MLP. The vector derived from the first token in the output from the point-wise MLP was used to compute the final output; the vector was used as input to a softmax or linear function depending on the given problem (classification or regression) to produce the final result. In this study, we used a simple point-wise MLP without Attention or Relation as a Baseline Model (Baseline) for comparison. Three layers of point-wise MLP were used to ensure that the parameter size is consistent with the other networks. For all networks, initial weight parameters were initialized using Glorot uniform distribution (Glorot and Bengio, 2010), and bias parameters were initialized by the null vector. The source codes used to generate all the networks in this study are available on the GitHub repository, github.com:yamada-kd/Relation.git.

#### 3.2.2. Performance of context processing

We have used the General Language Understanding Evaluation (GLUE) benchmark dataset to evaluate the ability of each neural network to process contextual information. It consists of a total of 11 test datasets of various types related to natural language processing (CoLA, SST-2, MRPC, STS-B, QQP, MNLI-m, MNLI-mm, QNLI, RTE, WNLI, and AX). It is the most well-known benchmark system for evaluating artificial intelligence dealing with contextual information (Wang et al., 2019). It should be noted that the AX dataset is referred to as a diagnostic dataset in the original paper. The predictors were grown using the provided training dataset, and the final predictor was obtained by early stopping with the patience set to five. Adam (Kingma and Ba, 2014) was used with default hyperparameter settings to update the parameters. Training data were fed into the networks as minibatches with a size of 256. Dropout was used with a dropout rate of 0.5 as the regularization method for the networks. The constructed predictor solved the test problem, and results were sent to the GLUE server to obtain GLUE scores. The description of scores for each test is given in the cited paper and is not explained here. Positional encoding similar to that of Transformer was implemented (Vaswani et al., 2017). Token embedding was done with a vector of 100 lengths, and GloVe was used as the initializer for embedding (Pennington et al., 2014).

#### 3.2.3. Computation time

The length of the longest sequence in the GLUE dataset is 467, while that in the IMDb dataset (Maas et al., 2011) and Reuters newswire classification dataset (Reuters) (UCI Machine Learning Repository, 1997) provided by TensorFlow (Abadi et al., 2015), and WikiText-103 (Merity et al., 2016) is 2,494, 2,376 and 6,231 respectively, which are very long. As mentioned above, the time complexity of Attention is *O*(*N*^2^), and that of Relation is *O*(*N*). We benchmarked the computation times on these datasets to determine the difference in actual computation time when using long input sequences. WikiText-103 includes pairs of a header and sentences in an instance. For the dataset, we conducted the binary classification task, where each method classifies whether the input sentences belong to the secondary level headers or the other level headers. The number of instances in the learning dataset of IMDb, Reuters, and WikiText-103 is 25,000, 8,982, and 304,092, respectively. We measured the time taken for the accuracy of the validation dataset to reach 0.8 consecutively 10 times.

#### 3.2.4. Statistical analysis

All the experiments in this study were repeated five times by changing the initial parameters randomly. The results display the computed mean values and standard deviations. The performance of each pair of methods was compared at a confidence level of 0.95 using the Steel-Dwass test.

## 4. Results

### 4.1. Performance to process context

Table 1 shows the results of GLUE-based prediction performance benchmarks. It can be observed that the prediction performance is not as good as that of existing state-of-the-art methods or complex structures such as Transformer. However, this is natural because the objective of this experiment was to verify if the Relation can process contextual information similar to Attention and not whether Relation can achieve high prediction performance when used for complex structures such as Transformer.

**Table 1:** Benchmark results with general language understanding evaluation (GLUE).

**Method**	**Avg**	**CoLA**	**SST-2**	**MRPC**	**STS-B**	**QQP**	**MNLI**	**QNLI**	**RTE**	**WNLI**	**AX**
***Parameter size: 64***											
Baseline	43.6	–0.380	59.6	**79.9**/66.5	13.7/14.2	16.4/**80.3**	38.2/38.2	53.2	50.4	55.8	0.460
	*0.705*	*0.850*	*1.01*	*0*/*0*	*1.07*/*1.56*	*8.09*/*1.90*	*0.464*/*0.286*	*0.365*	*0.653*	*6.15*	*0.802*
Attention	50.6	2.32	73.6	75.4/65.7	24.9/24.0	**51.6**/80.1	52.4/51.0	57.2	51.0	58.6	4.78
	*1.13*	*3.29*	*0.819*	*2.37*/*1.45*	*0.673*/*1.45*	*0.753*/*1.73*	*0.546*/*0.447*	*0.308*	*0.796*	*11.0*	*0.981*
Linear	52.7	8.92	79.8	79.2/**68.9**	25.2/25.9	49.3/75.8	54.9/53.4	57.1	51.3	**61.0**	7.12
	*0.942*	*1.45*	*0.869*	*1.32*/*1.11*	*1.66*/*1.63*	*0.540*/*0.623*	*0.316*/*0.567*	*0.517*	*0.581*	*7.66*	*1.82*
Relation	**53.5**	**10.8**	**80.6**	76.5/67.4	**28.3**/**27.3**	51.5/79.0	**55.4**/**54.5**	**58.0**	**51.8**	60.2	**9.74**
	*0.952*	*2.36*	*0.397*	*1.65*/*1.14*	*1.65*/*3.09*	*0.650*/*2.11*	*0.377*/*0.447*	*0.555*	*0.858*	*8.22*	*0.885*
***Parameter size: 128***											
Baseline	43.8	–1.24	60.2	**79.3**/66.0	14.0/15.3	23.8/78.1	38.2/38.5	52.9	51.0	54.7	1.02
	*1.11*	*1.37*	*1.01*	*1.32*/*1.13*	*0.832*/*1.91*	*10.8*/*2.78*	*0.277*/*0.391*	*0.292*	*0.656*	*10.5*	*1.65*
Attention	51.7	9.46	73.6	76.3/66.6	25.9/24.3	51.0/**79.2**	53.4/52.5	56.8	52.1	59.3	6.32
	*0.897*	*1.66*	*0.658*	*1.06*/*0.814*	*1.49*/*1.68*	*0.526*/*1.26*	*0.251*/*0.800*	*0.730*	*0.297*	*8.91*	*1.32*
Linear	**53.7**	9.78	80.0	77.9/**68.3**	28.4/29.1	50.0/76.7	**56.6**/55.2	56.5	**52.4**	**63.4**	**10.2**
	*0.589*	*1.44*	*0.777*	*1.52*/*0.876*	*2.51*/*1.71*	*0.483*/*1.08*	*0.344*/*0.439*	*0.439*	*0.936*	*3.03*	*1.48*
Relation	53.6	**10.6**	**81.2**	73.9/65.9	**31.2**/**30.3**	**51.1**/77.3	**56.6**/**55.8**	**58.4**	51.6	59.7	9.48
	*0.427*	*1.99*	*0.702*	*2.24*/*1.46*	*1.09*/*1.38*	*0.940*/*1.11*	*0.336*/*0.543*	*0.223*	*0.555*	*6.01*	*1.67*
***Parameter size: 256***											
Baseline	43.6	1.30	60.1	75.4/63.2	13.0/14.0	11.9/**80.7**	37.8/38.4	53.0	51.8	59.3	1.16
	*1.24*	*2.25*	*1.33*	*1.64*/*1.34*	*1.06*/*1.13*	*11.8*/*2.33*	*0.192*/*0.303*	*0.179*	*0.515*	*10.3*	*0.802*
Attention	51.9	8.28	74.2	76.1/66.4	25.3/24.2	**51.6**/80.4	54.0/53.0	57.2	52.4	59.9	6.84
	*1.44*	*3.76*	*0.339*	*1.03*/*0.760*	*2.94*/*3.24*	*0.292*/*1.33*	*0.288*/*0.487*	*0.650*	*0.270*	*8.83*	*1.98*
Linear	54.1	9.10	80.3	**78.0**/**68.5**	31.7/31.2	50.3/76.4	57.0/55.5	56.4	**52.9**	**64.4**	10.4
	*0.416*	*1.93*	*0.658*	*1.59*/*1.14*	*1.36*/*1.11*	*0.381*/*0.336*	*0.100*/*0.259*	*0.187*	*0.587*	*1.94*	*0.462*
Relation	**54.2**	**10.5**	**81.0**	74.9/66.3	**33.5**/**32.2**	51.2/76.7	**57.8**/**56.7**	**58.8**	52.6	59.4	**12.4**
	*1.06*	*2.05*	*3.61*	*3.61*/*2.35*	*1.46*/*1.24*	*0.581*/*1.07*	*0.342*/*0.182*	*0.497*	*0.432*	*5.25*	*1.50*

The number to the right of “Parameter size” denotes the depth of Attention, Relation, or MLP. “Linear” denotes Linear Attention. For MNLI, accuracy on MNLI-m and MNLI-mm are reported. For MRPC and QQP, accuracy and F1 are reported. For STS-B, Pearson and Spearman correlations are reported. For CoLA, Matthews correlation is reported. For all other tasks, accuracy is reported. The values in the upper and lower panel in each method represent the mean and absolute value of the standard deviation (italic value) based on five times the evaluation scores. The evaluation scores are scaled by 100. The best value in each block is highlighted in boldface.

The general language understanding evaluation score measures accuracy, correlation coefficient, or F1 score, and a high value indicates good performance. The GLUE benchmark consists of 11 evaluation items, the most important of which is AX. For the remaining 10, training and validation datasets are provided to train the predictors to make predictions on the test dataset; however, for AX, neither the training dataset nor the validation dataset is provided. AX is a problem that considers two sentences as input and classifies them into three classes. Since MNLI is a similar problem, we have used a predictor trained on this dataset to predict AX, which is completely independent of the other 10 datasets.

It was observed that the baseline model performed worse than Attention or Relation predictors on almost all datasets at all depths and very poorly on AX. Baseline is a method that does not consider any context of the input sequences. Network size does not have any influence on model performance since the size of each method at each depth is unified; the GLUE benchmark performs better when the context is taken into account. Hence, the inability of the baseline model to consider context may be the reason for its low prediction performance. The performance of networks with Attention and Linear Attention, including the value of AX improved with increasing depth. Furthermore, the prediction performance using these networks was better than that of Baseline because the context of the input sequences is taken into account. We conducted statistical analysis for the values of AX and Avg, which was also called the GLUE score. Consequently, the performance of Attention and Linear Attention was significantly better than that of Baseline on both criteria at all depths. Meanwhile, the performance of Linear Attention was significantly better than that of Attention on both criteria when the depth size was 128. Their performance seemed to be comparable to each other in other cases.

By contrast, the network with Relation also exhibited improved prediction performance depending on the depth size. The performance of Relation was significantly better than that of Baseline on both criteria at all depths according to the statistical test. Additionally, the performance of Relation was better than that of Attention on avg when the depth size was 128 and on AX when the depth size was 64 or 256. Furthermore, the performance of Relation was significantly better than that of Linear Attention on AX when the depth size was 256. No statistical significance was observed in other pairwise comparisons among the three methods. However, the prediction performance of Relation was inferior to Attention and Linear Attention in some cases on MRPC, QQP, RTE, and WNLI, although the difference was not statistically significant. The distinctive feature of these problems was that Baseline, which in principle could not process context information, performed comparably to the other methods. This means that when solving these problems, it is not so important for methods to process the context information, compared to the other problems. Therefore, it can be assumed that in addition to Baseline, all methods that can process the context information, such as Attention, Linear Attention, and Relation, would have only shown comparable prediction performance. Conversely, considering that Relation performed statistically significantly better than Attention and Linear Attention in some cases on the other problems that required methods to process context information. Relation may be a promising mechanism for processing contextual information. Taken together, we concluded that the performance of Relation was better than or comparable to Attention and Linear Attention on the datasets tested in this study. Thus, it is clear that contextual information can be processed by Relation in the same manner as Attention and Linear Attention.

### 4.2. Computation time

The results of evaluating the computation time of each method using the IMDb, Reuters, and WikiText-103 datasets are shown in Table 2. The calculation with Baseline, which consists of only point-wise MLPs, could not be completed because its accuracy did not increase depending on the progress in epochs. The accuracy using Baseline was approximately 0.5 on IMDb, 0.3 on Reuters, and 0.5 on WikiText-103 from the first epoch to the end of the computation. The Baseline exhibited decent prediction performance on some of the GLUE datasets. GLUE has numerous short input sequences, so it is likely that in some cases if certain tokens are used for prediction, accurate predictions can be made without taking the context into account. However, the sentences in IMDb, Reuters, and WikiText-103 are long and composed of a variety of words; hence, it is assumed that the correct answer could not be derived without considering the context.

**Table 2:** Computation time (s) of learning phase for IMDb, Reuters, and WikiText-103.

	**IMDb**		**Reuters**		**WikiText-103**	
**Method**	**Total time**	**Time/epoch**	**Total time**	**Time/epoch**	**Total time**	**Time/epoch**
***Parameter size: 64***						
Baseline	n/a	n/a	n/a	n/a	n/a	n/a
	*n/a*	*n/a*	*n/a*	*n/a*	*n/a*	*n/a*
Attention	316	15.3	952	4.35	44,100	1,100
	*13.8*	*0.0108*	*111*	*0.00920*	*1,600*	*1.73*
Linear	64.1	2.14	233	0.720	**1,270**	65.9
	*5.92*	*0.00656*	*50.8*	*0.00213*	*85.2*	*3.80*
Relation	**32.9**	**1.73**	**145**	**0.579**	1,410	**50.3**
	*0.103*	*0.00543*	*10.9*	*0.00727*	*34.8*	*1.24*
***Parameter size: 128***						
Baseline	n/a	n/a	n/a	n/a	n/a	n/a
	*n/a*	*n/a*	*n/a*	*n/a*	*n/a*	*n/a*
Attention	347	17.2	563	4.93	44,200	1,210
	*7.69*	*0.00800*	*29.0*	*0.00650*	*1,370*	*1.68*
Linear	107	3.73	172	1.27	**2,050**	108
	*3.04*	*0.0554*	*13.4*	*0.00249*	*67.6*	*3.56*
Relation	**54.1**	**2.85**	**115**	**0.971**	2,080	**81.1**
	*0.352*	*0.0185*	*7.07*	*0.0152*	*59.0*	*2.97*
***Parameter size: 256***						
Baseline	n/a	n/a	n/a	n/a	n/a	n/a
	*n/a*	*n/a*	*n/a*	*n/a*	*n/a*	*n/a*
Attention	431	22.2	445	5.65	51,000	1,540
	*11.7*	*0.0539*	*14.2*	*0.0360*	*1,110*	*1.58*
Linear	166	6.12	190	2.09	3,520	185
	*13.1*	*0.00934*	*9.36*	*0.0112*	*69.6*	*3.66*
Relation	**86.1**	**4.53**	**134**	**1.59**	**3,400**	**137**
	*0.0957*	*0.00505*	*3.89*	*0.0300*	*107*	*2.88*

Total learning time and time per epoch are presented. The number to the right of “Parameter size” denotes the depth of Attention, Relation, or MLP. “Linear” denotes Linear Attention. The values in the upper and lower panels in each method represent the mean and absolute value of the standard deviation (italic value) based on five times the evaluation scores. The best value in each block is highlighted in boldface.

The accuracies of Relation, Attention, and Linear Attention increased depending on the learning progress; based on this, we reconfirmed that Relation and Attention could process the context information properly. Among them, the network with Relation exhibited the best performance in terms of total computation time and computation time per epoch at all depths on IMDb and Reuters. According to the statistical analysis, the performance of Relation on the total computation time and computation time per epoch on these datasets is significantly better than that of both Attention and Linear Attention at all depths. The overall parameter size for each network is unified for each depth, meaning the size of the parameters does not affect the computation performance. As mentioned earlier, the time complexity for both Linear Attention and Relation against the sequence length is *O*(*N*). Nevertheless, the computation time of Relation is better than Linear Attention. It would be due to the following reason. The time complexity for Linear Attention is 2*Nd*^2^+3*Ndm* and that for Relation is *Nd*^2^+2*Ndm*+*Nd*, when the projection and depth of the input sequence are considered for calculation. Therefore, Linear Attention calculation requires *Nd*^2^+*Nd*(*m*−1) more computations than Relation. The difference in the computation load between Linear Attention and Relation is due to the difference in actual computation time in the experimental results. Whereas, when we used WikiText-103, which consisted of a longer sequence, the tendency changed; the total computation time of Relation was comparable to that of Linear Attention, though Linear Attention and Relation were significantly much better than Attention, according to the statistical test. The computation using Attention took a surprisingly long time, emphasizing the effect of differences in time complexity between *O*(*N*) and *O*(*N*^2^), and the usefulness of Relation and Linear Attention on long sequence processing was reconfirmed. Comparing to Linear Attention, Relation required more epochs to achieve good prediction performance at depth of 64. Whereas at depth of 128, there was no statistical significance, and at depth of 256, the total computation time of Relation was statistically significantly better than that of Linear Attention. These results showed that the performance of Linear Attention and Relation on the total computation time was comparable. Depending on a problem and depth, either Linear Attention or Relation would be able to reach the correct answer in less computation time than Relation or Linear Attention; in actual use, a better method should be adopted depending on the problem. Whereas the computation time per epoch of Relation was statistically significantly better than that of Linear Attention at all depths. Taken together, Relation can achieve the same prediction performance as Attention in a shorter duration and Linear Attention in a comparable or shorter duration.

## 5. Discussion

In this study, we devised a mechanism that considers context information and compared its performance to networks with Attention and simple MLPs without context processing capability. The attention mechanism has been used in various networks, including Transformer. The motivation behind developing the proposed method was to improve the computation time for Attention. This improvement affects various fields, such as natural language processing and image processing. Therefore, extensive research has been conducted on the improvement of computation time for Attention (Tay et al., 2020). The time complexity of Attention was *O*(*N*^2^) and that of Linear Attention was *O*(*N*). Thus, Linear Attention exhibited better performance from the perspective of computation time, and our goal in this study was to create a mechanism with the same level of computational complexity and prediction performance as Linear Attention. Improving time complexity is not merely a benefit of reducing the time required for learning. It is undeniable that we can further improve the performance of the final artificial intelligence in the same computational time by making the network structure more complex instead of reducing the computational time if the method with reduced time complexity has the same context processing capability as Attention. From this perspective, Relation is considered to be a useful method for context processing.

Over the years, the amount of data available has increased exponentially. The real world contains a wide variety of data, including natural language, images, biological strings, economic time series data, and contextual information. The Relation can be useful for dealing with such data. In this study, we implemented a simple approach in which the information of each token in the input sequence was aggregated into a single vector by computing an element-wise mean value of each vector projected from each token in the input sequence. The aggregated information was then returned to the network corresponding to each token; that is, most of the computation in Relation consists of MLP, which would be surprising for some researchers. However, the idea of using MLP alone to process context is not novel. Like RNNs, MLPs can process context information because they can estimate all nonlinear relationships by the universal approximation theorem. One of their implementations is MLP-Mixer, which consists of MLPs alone to process context information (Tolstikhin et al., 2021). Since the goal of this study was to improve the time complexity of Attention, we did not consider MLP-Mixer for comparison, which has a time complexity of *O*(*N*^2^). However, it would be useful to compare Relation with other methods that use mechanisms other than Attention. In addition to MLP-Mixer, for example, there is another method that can process context information, the Sigma-if neural network, which is a contextual generalization of MLP (Huk, 2012). A comparison with such a method that enables contextual processing through a mechanism other than Attention will help further clarify the pros and cons of Relation. In the future, we intend to continue our efforts to find new methods because there could be better ways of capturing the entire contextual information than Relation or other methods.

Benchmarks with GLUE, a benchmark system for natural language processing, and benchmarks on datasets containing long sequences revealed that Relation can process contextual information with improved computation time. The objective of this study was to verify if Relation could process context information faster than Attention and not if it could achieve high prediction performance when used with complex structures such as Transformer. In other words, a limitation of this study is that it is unclear whether Relation will perform efficiently when used as a component in a larger network structure such as Transformer. This study only revealed that Relation can process contextual information that a simple MLP (Baseline) could not process; however, it can process as much information as Attention in a small network structure. Furthermore, other aspects should be verified. In this study, we used common parameter settings and methods when training neural networks including Relation and other neural networks used for comparison. For example, the pseudo-random number generator (PRNG) used to initialize the neural network parameters was the Philox algorithm (Salmon et al., 2011), which is the default PRNG in TensorFlow. However, it has been reported that PRNG may have a particular impact on neural networks that handle contextual information (Huk et al., 2021) and thus selecting and using a suitable PRNG is an important factor to consider in the future. In the future, we will analyze the effectiveness of Relation on large networks in addition to these improvement studies. The possibility of its use in various fields will increase if the effectiveness of Relation in large networks is clarified; however, this is not to say that there is no value in using Relation in the present situation. Since Relation can process context information, at least in small structures, and is computationally superior to Attention, it may be a viable alternative to Attention in situations where large amounts of data must be processed within a limited time frame.

## Data availability statement

Publicly available datasets were analyzed in this study. This data can be found at: https://gluebenchmark.com/.

## Author contributions

The concept of the study was conceived by KDY. All the authors conducted the analysis. All the authors made contributions to writing the manuscript and approved the final version of the article.

## Funding

This work was supported in part by the Top Global University Project from the Ministry of Education, Culture, Sports, Science, and Technology of Japan (MEXT) and the Research Support Project for Life Science and Drug Discovery [Basis for Supporting Innovative Drug Discovery and Life Science Research (BINDS)] from AMED under Grant No. JP22ama121019.

## Conflict of interest

The authors declare that the research was conducted in the absence of any commercial or financial relationships that could be construed as a potential conflict of interest.

## Publisher's note

All claims expressed in this article are solely those of the authors and do not necessarily represent those of their affiliated organizations, or those of the publisher, the editors and the reviewers. Any product that may be evaluated in this article, or claim that may be made by its manufacturer, is not guaranteed or endorsed by the publisher.
